# Cdc14b: A New Player in Aging

**DOI:** 10.18632/aging.100332

**Published:** 2011-05-26

**Authors:** Zhubo Wei, Pumin Zhang

**Affiliations:** Department of Molecular Physiology and Biophysics at Baylor College of Medicine, One Baylor Plaza, Houston, TX 77030, USA

Cdc14 encodes a dual specificity protein phosphatase essential for mitotic exit. There are two Cdc14 isoforms (a and b) in vertebrates. Cdc14a localizes to the centrosome and Cdc14b localizes to the nucleolus [1]. The two mammalian Cdc14 isoforms have been speculated to exert essential mitotic functions, given the role of budding yeast ScCdc14 played in the cell cycle and the fact that both human CDC14A and CDC14B could rescue the mitotic exit defects of ScCdc14 mutants [2, 3]. However, it is apparent now that neither Cdc14a nor Cdc14b is required for mitotic exit, not even for the cell cycle [4-6]. Instead, both of them were shown to be involved in DNA damage response [4-7]. It is unclear how these two paralogous phosphatases are involved in DNA damage repair. It is imaginable that they are probably required to dephosphorylate some substrates that participate in the repair process.

When Cdc14b-deficient mice were left to age, they develop cataracts and kyphosis at early ages [6], suggesting a role of Cdc14b in preventing the early onset of aging. Although this premature aging phenotype is consistent with the role of Cdc14b in DNA damage repair, we could not exclude the possibility that Cdc14b is involved in other cellular processes that are more important in preventing aging. Thus, we noticed a synergistic effect on aging when Cdc14b deficiency was combined with Cdh1 haploinsufficiency (Wei and Zhang, unpublished observation). Cdh1 is the adaptor protein for the E3 ubiquitin ligase APC (anaphase promoting complex). In budding yeasts Cdh1 is downstream from Cdc14 [8], which may also be true in mammals. However, there is no evidence suggesting a role of Cdh1 in DNA damage repair. Thus, it is possible that Cdc14b's function in aging is through Cdh1 in processes not related to damage repair. It will be interesting to see if Cdc14a also plays a role in aging.
